# Recursive dynamic functional connectivity reveals a characteristic correlation structure in human scalp EEG

**DOI:** 10.1038/s41598-021-81884-3

**Published:** 2021-02-02

**Authors:** Siddharth Panwar, Shiv Dutt Joshi, Anubha Gupta, Sandhya Kunnatur, Puneet Agarwal

**Affiliations:** 1 Department of Electrical Engineering, Indian Institute of Technology, Delhi, New Delhi, 110016 India; 2 Department of Electronics and Communication Engineering, Indraprastha Institute of Information Technology, New Delhi, 110020 India; 3 Arghya Bioinformatics, New Delhi, India; 4 Max Super Speciality Hospital, Saket, New Delhi 110017 India

**Keywords:** Network models, Network topology, Data processing

## Abstract

Time-varying neurophysiological activity has been classically explored using correlation based sliding window analysis. However, this method employs only lower order statistics to track dynamic functional connectivity of the brain. We introduce recursive dynamic functional connectivity (rdFC) that incorporates higher order statistics to generate a multi-order connectivity pattern by analyzing neurophysiological data at multiple time scales. The technique builds a hierarchical graph between various temporal scales as opposed to traditional approaches that analyze each scale independently. We examined more than a million rdFC patterns obtained from morphologically diverse EEGs of 2378 subjects of varied age and neurological health. Spatiotemporal evaluation of these patterns revealed three dominant connectivity patterns that represent a universal underlying correlation structure seen across subjects and scalp locations. The three patterns are both mathematically equivalent and observed with equal prevalence in the data. The patterns were observed across a range of distances on the scalp indicating that they represent a spatially scale-invariant correlation structure. Moreover, the number of patterns representing the correlation structure has been shown to be linked with the number of nodes used to generate them. We also show evidence that temporal changes in the rdFC patterns are linked with seizure dynamics.

## Introduction

Recent advancements in brain mapping techniques and the availability of inexpensive computational resources have enabled extensive exploration of neurophysiological data. One of the prominent areas of interest has been the study of functional connectivity (FC) where statistical dependencies between spatially distinct regions of the brain are identified^1^. Functional connectivity patterns have led to the identification of large-scale cerebral networks that reveal the various ways in which the brain is organized^2,3^. Complex network analysis has been widely employed to characterize these networks through graph-theoretic measures^4^. This combination of network science with cognitive neuroscience has given rise to the new discipline of network neuroscience^5^ that has allowed a deeper understanding of brain function with the use of both fMRI and electrophysiological data. Studies have revealed that the architecture of functional networks embeds information of an individual’s state of consciousness^6^, learning progression^7^, intelligence^8^ and age^9^. Moreover, biomarkers for many disorders such as dementia^10,11^, Parkinson’s disease^12,13^, multiple sclerosis^14,15^, depression^16^ and epilepsy^17,18^ have also been obtained.

In earlier work, calculations were performed over the entire neurophysiological activity data to obtain static measures for FC, which ignored the time-varying aspects of the connections. The recognition of the potential of dynamic functional connectivity (dFC)^19,20^ for understanding brain networks led to the development of several techniques leveraging the modulating nature of the activity data^21–23^, with the most popular among them being the method of sliding window^24^. Here, a fixed length window steps through the activity data and generates dynamic connectivity time series by sequentially computing a connectivity measure in each window. The fluctuations captured in the dynamic connectivity time series are then summarized by various means such as k-means clustering^25^, principal component analysis (PCA)^26^ or independent component analysis (ICA)^27^. Despite a wide-ranging effort, the complexity of brain networks continues to provide tremendous potential for further exploration of functional connectivity through novel means.

Several FC measures have been proposed^28^, with Pearson correlation coefficient being the most direct measure of FC in the sliding window framework^29^. Although it is one of the most commonly used measures of statistical coupling^30^, Pearson correlation is limited to lower order statistics. We propose recursive dynamic functional connectivity (rdFC) to examine the temporally rich dynamics of EEG^31^ through higher-order correlations. The proposed technique was inspired by principal component analysis (PCA) as applied to multivariate dynamic connectivity time series data where correlation is the measure for dFC^26^. Here, computationally one is required to calculate correlations of dynamic correlations. Taking a cue from this, a higher-order dynamic connectivity time series can be computed by applying a sliding window on a previously obtained dynamic connectivity time series as if it was activity data. When done recursively, this process gives a series of higher-order dynamic correlations time series for which static correlation coefficients are computed as summary measures. These static correlation coefficients are collectively visualized and seen as a multi-order connectivity pattern.

Taking three electrodes at a time, we discover a common correlation structure expressed via three connectivity patterns in EEGs of subjects irrespective of their age, neurological health, vigilance states, location of the electrodes on the scalp, and morphological features in their EEG. The three distinct patterns are shown to be equivalent and can be transformed from one to another by permuting the sequence in which the nodes are processed. The discovery of three connectivity patterns representing the underlying correlation structure is demonstrated to be an outcome of the choice of using three nodes for generating the connectivity patterns. Additionally, the three connectivity patterns are shown to be scale-invariant as they are observed for a range of mutual spacing among the triplet of electrodes. Though the exact connectivity strength varies vastly within and across EEG records, the widespread presence of this correlation structure suggests that a characteristic relationship between various connectivity orders persists across human scalp EEGs.

## Results

### rdFC reveals three equivalent connectivity patterns of one underlying correlation structure

**Figure 1:**
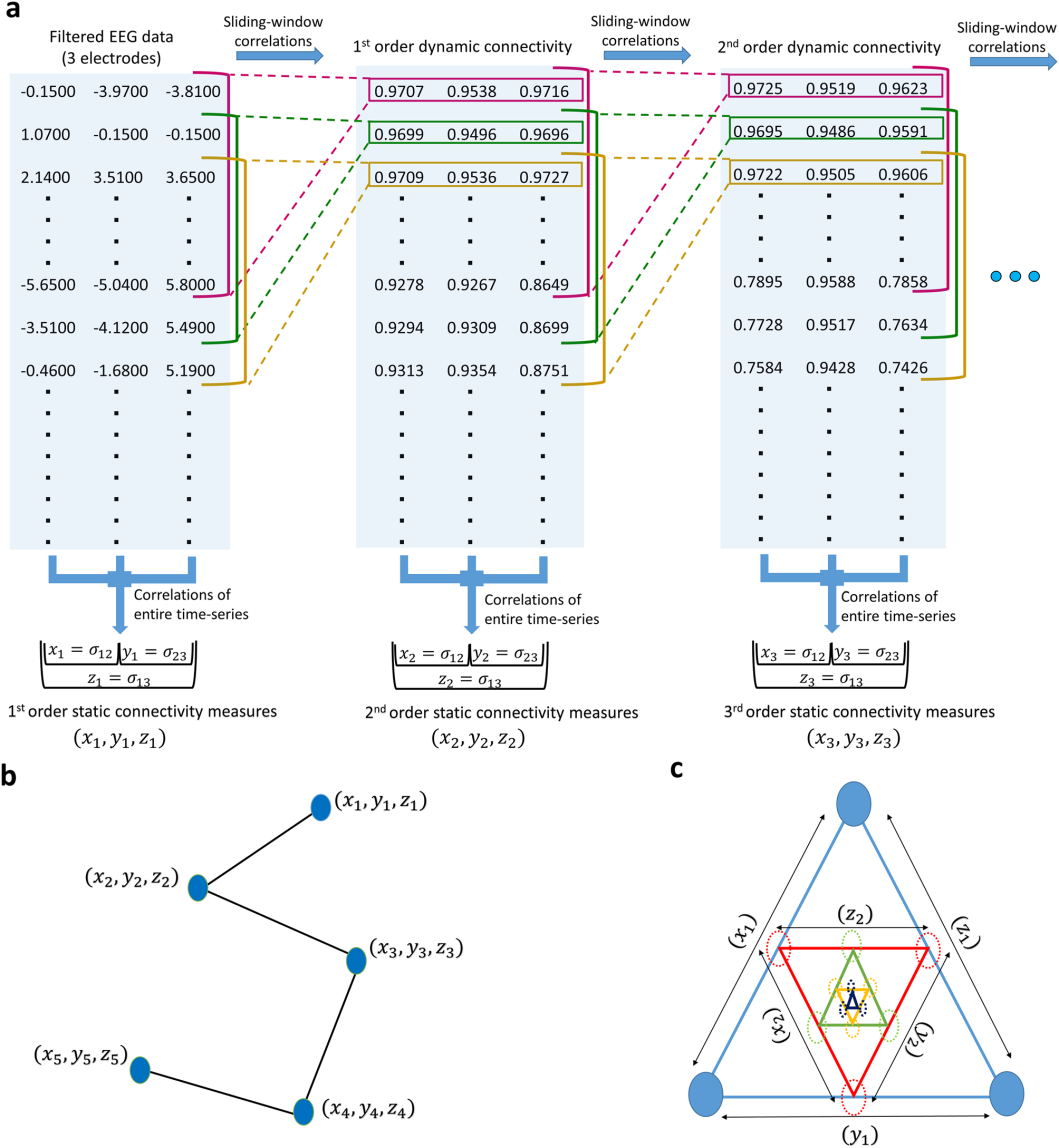
Computation of recursive dynamic functional connectivity of a triplet of electrodes. (**a**) The band-pass filtered EEG data of a set of three electrodes is processed using a sliding window to generate the 1st order dynamic connectivity time-series. The same sliding window is applied again on the 1st order dynamic connectivity time-series just obtained to produce the 2nd order dynamic connectivity time-series. The process of recursively applying the sliding window is carried out until the 4th order dynamic connectivity time-series is obtained, which is also the 5th set of time-series when including the original EEG data. In the second step, each of the five sets of time-series are summarized by taking correlation coefficients over their entire duration to compute three static connectivity measures at each order. (**b**) The three static connectivity measures computed at each order are then assigned to a coordinate value of a three dimensional point representing that order. Finally, all the five points corresponding to the five sets of trivariate time-series are connected with line segments to give a 5-point recursive dynamic connectivity pattern in a 3-dimensional space. (**c**) The multi-order connectivity of a triplet of electrodes can also be viewed as a hierarchical graph. Each triangle represents a particular order with the edges of the triangle representing the static connectivity measures. For the first order (outermost triangle) the nodes are the three electrodes used for rdFC analysis. The dynamic connectivity of these electrodes is seen as activity of pseudo nodes (dotted circles) that are connected by second order static connectivity measures. As we traverse inwards subsequent higher order static connectivity is represented as edges that connect their respective higher order pseudo nodes.

Recursive dynamic connectivity analysis (shown in Fig. 1) was originally performed to study long term continuous EEG records of epilepsy patients. Three distinct 5-point rdFC patterns, obtained for a set of three electrodes (triplet), were repeatedly observed while exploring these EEGs irrespective of the patient, location of electrodes, and time of the recording. This study was initiated to explore the presence of these three connectivity patterns across a much larger spectrum of scalp EEGs for electrode locations shown in Supplementary Fig. S1. A 5-point rdFC pattern is generated by computing three correlation coefficients each for five orders of connectivity, and then assigning them coordinates so that the set of correlation coefficients at each order can be represented as a single point in a 3-dimensional Cartesian coordinate system (see “Methods” section and Fig. 1b). The choice of which correlation coefficient gets assigned to which coordinate (*x*, *y*, *z*) is arbitrary but remains fixed in the algorithm. This assignment can be changed by permuting the sequence in which the algorithm receives the electrode data. For example, the sequence (C3,T3,T5) computes the same correlation coefficients as (C3,T5,T3), but the coordinate representations of the points in their respective 5-point pattern are entirely different because the coordinates of each point are in a different sequence (see Supplementary Table S1). There are P(3,3) = 6 sequences in which a triplet can be arranged. Each of these six sequences is referred to as a permutation, such as the two examples above. Each of these six permutations has its own rdFC pattern.

**Figure 2:**
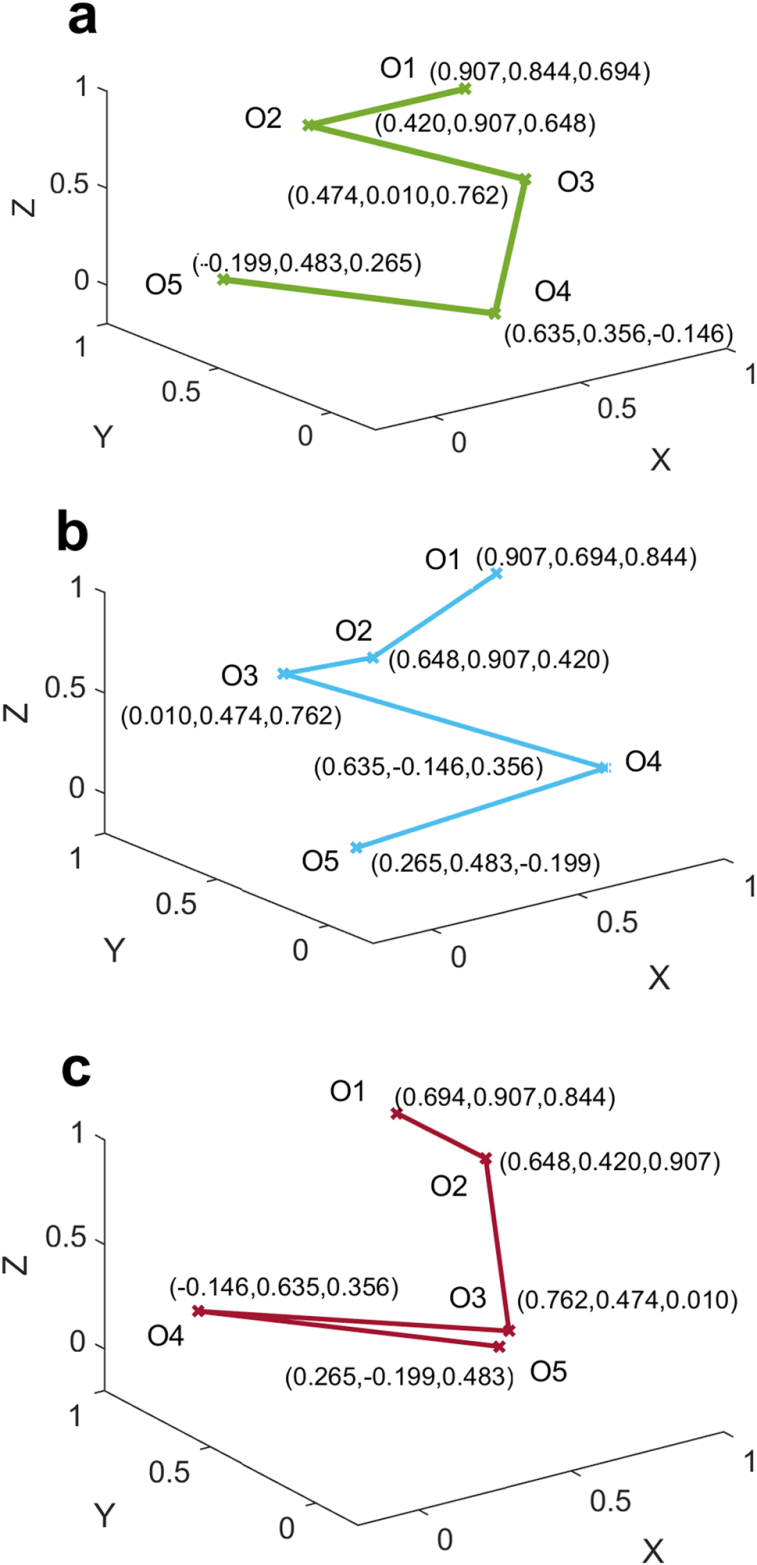
The three reference rdFC patterns. **a** Reference 1 **b** Reference 2 **c** Reference 3.

To study the prevalence of the three connectivity patterns, we first had to obtain three references that were representative of these patterns. Five-minute long data epochs of several triplets that were free of artefacts were identified in the corresponding author’s EEG and rdFC patterns of all six permutations for each of these epochs were computed. Remarkably, patterns that were representative of the three observed in our preliminary study of epilepsy patients were found within the six permutations of the same 5-min epoch of a single triplet, which were then chosen as references. The fact that these three rdFC reference patterns were generated from identical data just by reordering the sequence of electrodes indicates that they represent the same underlying correlation structure. We see this in Fig. 2 where the three coordinates of the points representing each order of connectivity are same across the three reference patterns and only their sequences are permuted. In fact, these three references are mathematically equivalent modulo the permutation operation performed on the electrode sequence prior to carrying out the rdFC analysis. To identify this correlation structure across different types of EEGs we matched its three representative reference patterns with patterns obtained from data sets described in the Methods section. The highest match score a pattern can get using the pattern matching scheme is 4, while a score greater than 2.65 qualifies it as a statistically significant match (see “Methods” section).

**Figure 3:**
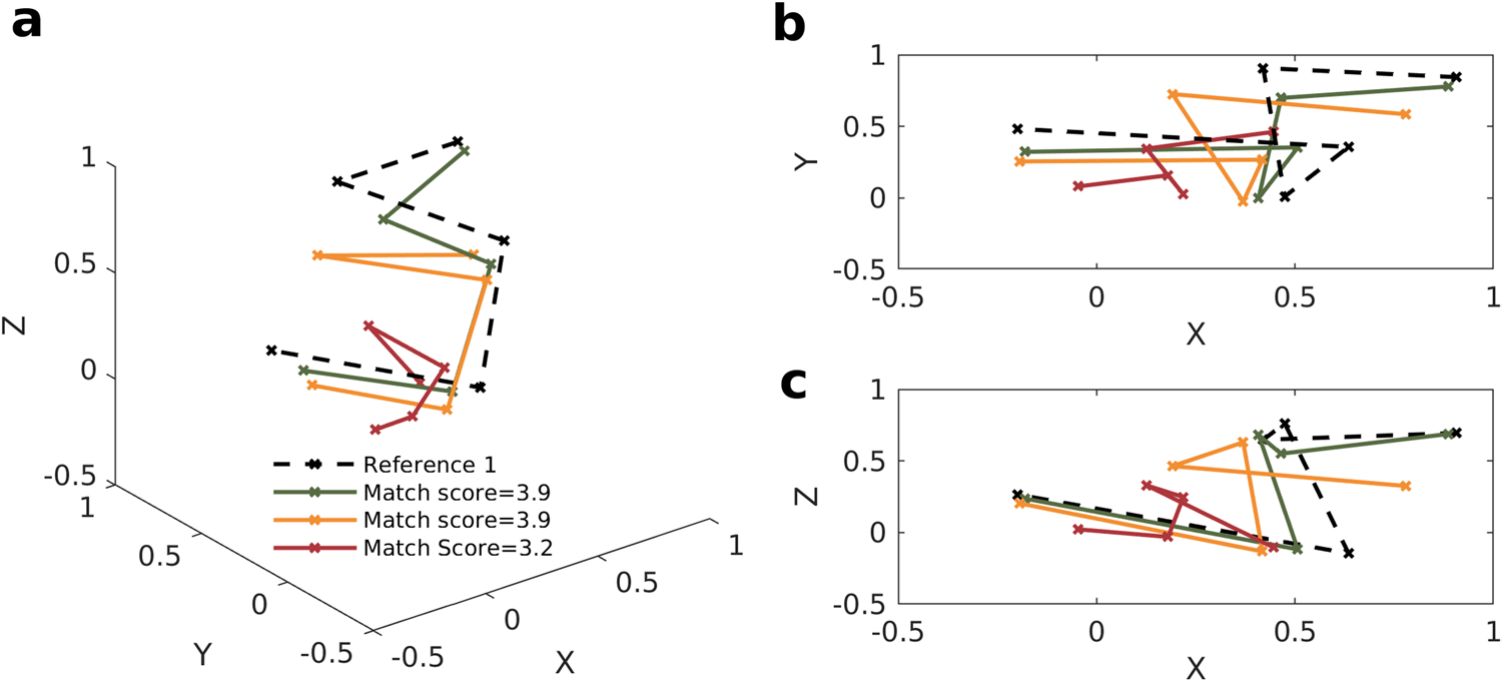
Location and scale (pattern size) invariance of the rdFC pattern matching algorithm. (**a**) Three sample rdFC patterns of different sizes and at different locations that give a statistically significant match score with reference 1. The three patterns were obtained from three different triplets in the EEG of a subject who was part of a past study^45^. All the 3-dimensional patterns have been projected in the (**b**) X–Y and (**c**) X–Z plane for a precise visual comparison of their slopes.

The pattern matching scheme was designed to be scale (pattern size) and translation invariant because we were looking to match the shape, and not the size and location of the patterns since the latter understandably varied. In Fig. 3, two patterns in different locations give the same high match score of 3.9. A pattern much smaller and distant from the reference pattern gives a lower match score of 3.2. This drop in the score is due to the greater differences in the shape of this pattern with the reference, and not its size and location. The differences in size and location of these patterns mean that the correlation coefficients computed across orders of dynamic connectivity vary substantially in the four patterns depicting them. However, the relationships between different orders of connectivity, governed by the slopes of the lines connecting the five points, stay relatively invariant and match those of the reference pattern. It is these slopes that determine the characteristic correlation structure (see Fig. 3b,c).

### The correlation structure is characteristic of scalp EEG and is spatially scale-invariant

**Figure 4:**
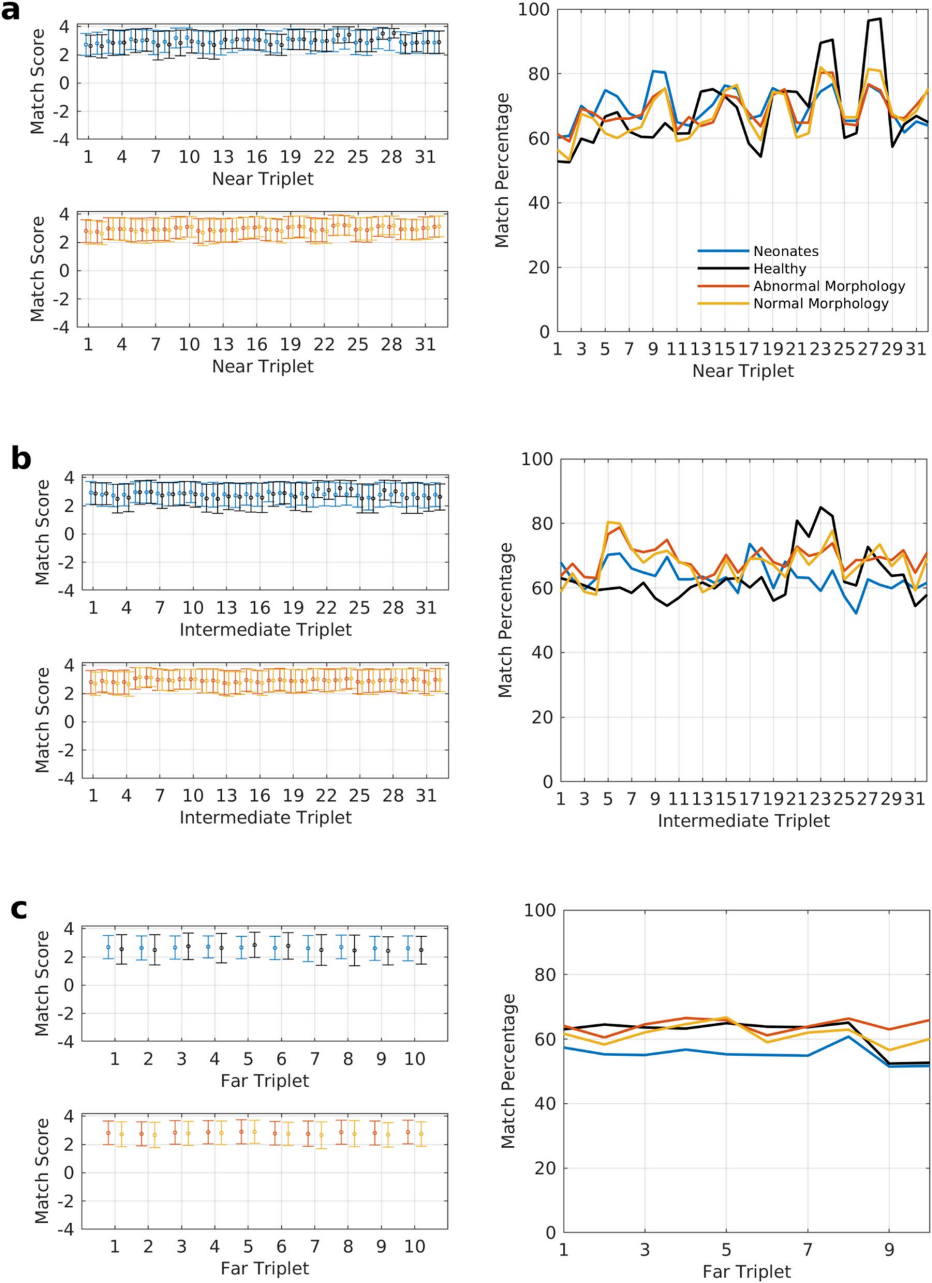
Match scores and match percentages for near, intermediate and far sets of triplets for data sets 2–4. The triplets have been assigned to their respective sets based on the mutual separation of the electrodes within a triplet. Each triplet in the three sets is represented by a number given in Supplementary Table S2. Consecutive odd and even numbered triplets are mirror images of each other in the two opposing cerebral hemispheres. The error bars represent 1 standard deviation. (**a**) Match scores and percentages for triplets in the near set. The triplets are arranged such that traversing towards the right on the x-axis corresponds to moving from the frontal portion of the scalp to the back. (**b**) Match scores and percentages for triplets in the intermediate set. With 2410 EEG records of 2356 subjects in the three data sets, 32 triplets for each record, and six permutations per triplet, (**a**,**b**) represent statistics of 462,720 connectivity patterns each. **c** Match scores and percentages of 144,600 connectivity patterns in the far set with 10 triplets.

A collection of diverse EEG signals is needed to investigate the universality of the correlation structure embedded in the reference connectivity patterns. The first place one encounters distinct EEG features is across the scalp of individual subjects. It is well known that different EEG rhythms are seen at specific locations on the scalp^32^, with the posterior basic rhythm in the parieto-occipital region of the cortex being the most prominent^33^. We studied nearly the entire scalp of all the subjects via three sets of triplets of varying mutual separation (see Supplementary Table S2). For addressing cross-subject variations in EEG, we obtained EEGs of 2356 subjects vastly differing in age and neurological health, with diverse morphological features exhibited in their EEG recordings (see data sets 2–4 in Table 2). Figure 4 plots the mean match scores and the percentage of connectivity patterns that exceed the statistically significant match score of 2.65 at each triplet location for these data sets. The mean scores were above the match threshold of 2.65 in most cases with scores in the healthy group being slightly lower, most likely due to the additional passband noise that was visually observed here in comparison with the other data sets. The match percentage is always greater than 55% for all three sets of triplets, irrespective of location on the scalp and data set.

The reference patterns were obtained from a triplet belonging to the near set. However, the overall match percentages of the patterns in all the data sets remain comparably high across all the three sets of triplets (see Table 1). The connectivity strengths are expected to vary substantially as the separation between the electrodes changes across the three sets of triplets. Still, we observe that the correlation structure embedded in the reference patterns is preserved remarkably well beyond the near set of triplets, signifying its spatial scale-invariance. We also explored match scores within individual EEG records for the near set of triplets. Among the 2410 records, there were only 110 with fewer than 50% of their patterns matching the reference patterns. Among these 110, we could find another EEG epoch in the record having a match percentage greater than 50% for all but 5 subjects. The cause for low match percentages was often EEG signal corruption, and a longer EEG recording for these 5 subjects would have likely yielded an epoch with a greater than 50% pattern match.

**Table 1:** Overall statistics for data sets 2–4 across all the three sets of triplets.

Dataset	Patterns matching reference patterns (%)			Triplets with no patterns matching (%)			Patterns predicted successfully from first order correlation coefficients (%)		
	Near	Intermediate	Far	Near	Intermediate	Far	Near	Intermediate	Far
Neonates	69.5	63.4	55.4	5.7	7.0	14.9	84.2	82.5	77.4
Healthy	68.0	63.3	61.7	4.7	1.0	2.8	89.6	85.5	69.5
Abnormal	68.8	69.1	64.2	5.7	3.9	6.1	88.3	88.5	82.6
Normal	67.7	67.5	61.4	7.3	4.7	7.2	87.6	87.8	80.1

The extrema of the curves in Fig. 4 depicting match percentages for specific locations on the scalp follow similar trends across data sets. This implies that there are region-specific variations in the rdFC patterns that extend across scalp EEGs. Equally interesting is the paired nature of these extrema in the near set of triplets. The grid lines in Fig. 4 are aligned with odd numbered triplets, and the even numbered triplets that follow them are their mirror images in the opposite cerebral hemisphere. The match percentages often rise and drop with the grid lines and stabilize for the following triplet in Fig. 4a. This suggests that local connectivity patterns, as captured by the near set of triplets, closely follow each other in the symmetrically opposite regions of the brain in the two corresponding cerebral hemispheres.

### The three equivalent patterns are equally prevalent

**Figure 5:**
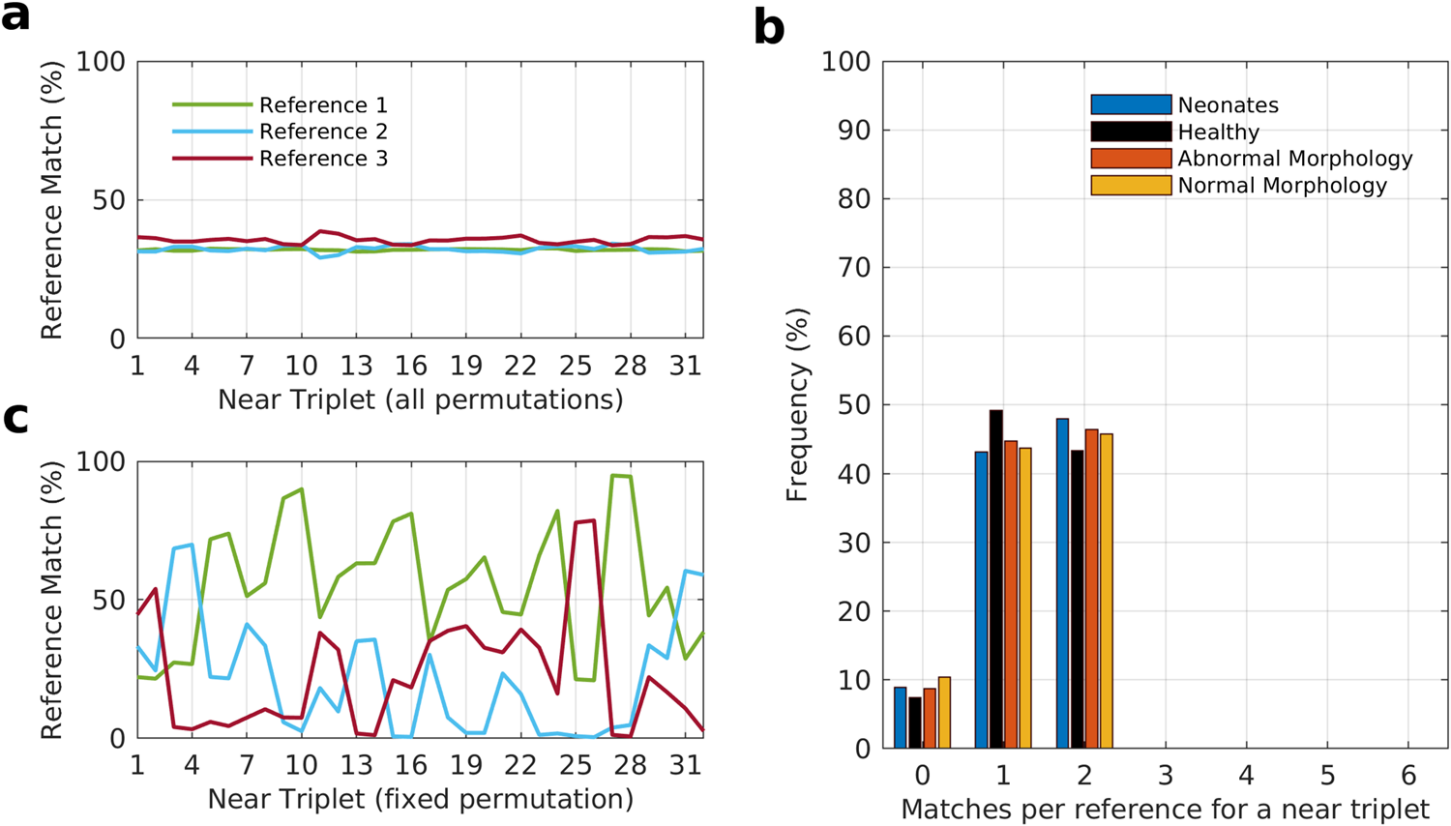
The prevalence of the three reference patterns relative to each other in the near set of triplets. (**a**) The percentage frequency with which each of the three references are observed across all permutations of a triplet (near) in data sets 2–4. The connectivity patterns of all six permutations at each triplet location that gave a statistically significant match were selected from the three data sets. The particular reference out of three that each pattern best matched with was noted and is shown as percentage frequency for all the three data sets. (**b**) Histogram for the number of permutations, among the six available per triplet, that matched with each of the three references. All three references are represented in this single histogram. A reference can match with anywhere between zero to all six permutations of a triplet. The histogram was constructed for a total of 77,120 triplets across all EEG records in data sets 2–4. (**c**) The percentage frequency with which each of the three references is observed in a fixed permutation of a triplet (near) across data sets 2–4. A particular permutation out of the six available for each triplet was arbitrarily chosen and kept fixed, unlike **a** where all six permutations were considered.

At this point, we know that the three equivalent representations of the underlying correlation structure can be observed across EEG records. However, we don’t know if all three representations are expressed equally. To address this question, we studied the relative prevalence of the three reference patterns in the near set of triplets. The near set was chosen because the references were also created from a triplet in the near set. First, we picked all the patterns that matched the references in data sets 2–4, i.e., those whose statistics are shown in Fig. 4a. We then noted which of the three reference patterns, in particular, each of the selected patterns matched. We can see in Fig. 5a that each reference contributes nearly equally (33%) in all the successful matches, implying that none of the three references is particularly dominant. The prevalence of the three references is comparable not just overall, but also at specific triplet locations across the scalp.

So far, our focus has been the connectivity patterns of individual permutations. To further explore the symmetry in the prevalence of the three references we looked at how they related to the overall collection of permutations of a triplet. As we know, each triplet generates six different permutations with corresponding rdFC patterns. We define a match vector with three indexed entries. Each entry represents the number of permutations of a single triplet that match with each of the three reference patterns. Theoretically, the three numbers in the vector can take any value between zero and six but they will always sum up to a maximum value of six. For example, the match vector [112] indicates that reference 1 and 2 found only one match each, while reference 3 matched with two of the six permutations of a triplet. Although, in this case, there is an asymmetry in the number of permutations each reference pattern matches with across a triplet, the symmetry is not entirely lost as none of the references match with more than a third, i.e., two out of six, of the available permutations of a triplet. Figure 5b shows the distribution of the entries within these match vectors for all the triplets in the near set for each data set. Strikingly, not one of the 77,120 triplets in all the data sets had more than two permutations match one of the references, giving further evidence that these three references are equally represented among the rdFC patterns.

Only around 10% of all triplets had a 0 element in the match vector, i.e., when none of the permutations of a triplet matched a particular reference (see Fig. 5b). Of all these cases, nearly two-thirds were contributed by triplets where all the three elements in the match vector were 0, i.e., none of the permutations matched any of the three references (see Table 1). In other words, if a triplet did not have a permutation matching with one of the three reference patterns, the chances are high none of its six permutations will match with any of the three references. All this evidence points to the fact that not only are the three reference patterns equivalent manifestations of the same underlying correlation structure, but they are expressed with near-equal probability.

### Number of connectivity patterns observed are linked to the number of electrodes used for pattern generation

**Figure 6:**
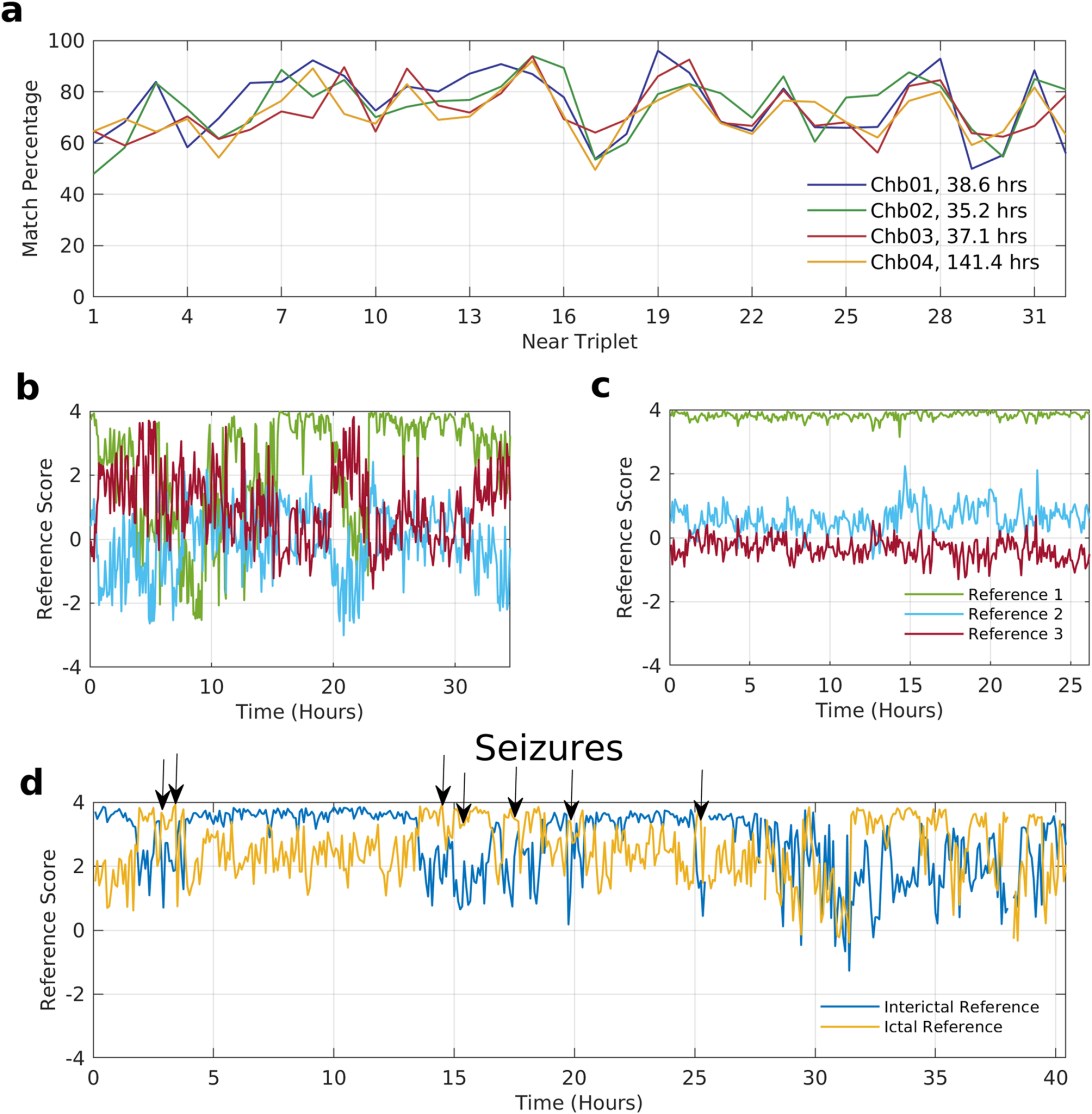
Temporal prevalence and evolution of rdFC patterns in data set 5. (**a**) Match percentage of first four subjects in data set 5 across all six permutations of each triplet. The patterns for each subject are obtained from each successive 5-min epoch of the entire EEG record of the respective patient. All the four EEG recordings are more than 24 h in duration, with record Chb04 spanning several days. Match scores for a specific permutation chosen for each triplet location with respect to all the three reference patterns when (**b**) there are regular fluctuations in the scores and (**c**) when the scores remain steady throughout the period of the recording. (**d**) Match scores of a specific permutation for a triplet location where seizure linked transitions in the rdFC patterns occur. The scores are with respect to two references that were obtained from an EEG epoch just before the third seizure (ictal reference) and an EEG epoch four hours prior to the third seizure (interictal reference).

Since the connectivity pattern observed can switch to a different one when a different permutation of the same triplet of electrodes is chosen, a question that naturally arises is whether a particular permutation is tied to a specific connectivity pattern. To answer this, we fixed the permutation (the sequence of electrodes) and looked at whether the same reference pattern appeared across subjects, and across time for the same subject. We learn that even when a particular permutation is chosen for a triplet, any of the three reference patterns can be observed across subjects (see Fig. 5c). The same is true when one tracks rdFC patterns across time for a fixed permutation of a subject. Figure 6b shows that while reference 1 gives a statistically significant match for a permutation for most of the recording period, it is replaced by reference 3 around the 5th and 22nd hour as the dominant rdFC pattern. So while patterns get switched by changing the permutation, a permutation is not bound to a particular reference pattern.

In previous sections changes in the connectivity patterns were an outcome of a computational manipulation, i.e., change in the sequence in which electrode data was processed. In Figs. 5c and 6b, the sequence of the electrodes is kept fixed and the observed variations in connectivity patterns are being driven by the underlying neuronal dynamics. Yet the geometric effects are the same in both scenarios because the pattern switching is occurring between the same set of reference patterns. This means that the principle determining which of the three reference patterns will be observed transcends both of the above individual governing factors. With this inference, our attention was drawn to the specific values of the correlation coefficients in the patterns. We discovered that the probability of a particular pattern matching with one of three reference patterns was strongly determined by which coordinate was carrying the lowest correlation coefficient value at the first order of connectivity, i.e., the first point of the 5-point pattern.

Based on our choice of coordinate assignment, we observed that when the lowest correlation coefficient of the first point of the 5-point connectivity pattern was at *x* coordinate the pattern matched with reference 3, when at *y* coordinate it matched with reference 2, and with reference 1 when at *z* coordinate. To test this hypothesis, we took all the patterns that matched the references from all the data and triplet sets and selected the ones that matched exclusively with only one of the three reference patterns. More than 90% of all the matching patterns belonged to this category. The shape of these patterns was predicted just by identifying the smallest coordinate of the first-order connectivity point and no knowledge of the other four higher order connectivity points was used. The prediction was successful in nearly 80% of the cases, except for a drop in case of neonates and healthy subjects for the far set of triplets (see Table 1).

In about 10% cases the patterns matched two references simultaneously, which were excluded in the above analysis of pattern prediction. In these patterns, instead of a clear lowest coordinate value, there were two closely spaced low valued coordinates. Consequently, the reference patterns corresponding to both of the two smallest coordinate values gave a high match score. The differences between the two lowest coordinates are significantly higher (p «0.001) among the set of patterns that match only one reference as opposed to those that match two references when comparing them within each set of triplets for all the data sets (see Supplementary Table S3). The lowest median difference between the two smallest coordinates in the former group across triplet and data sets is 0.07 (left half of Supplementary Table S4), while the highest median difference for the same in the latter group is 0.06 (right half of Supplementary Table S4), showing a decisive shift between the two sets of distributions.

Above we provided evidence that the observation of a particular pattern out of three reference patterns is dependent on which of the three coordinates of the point corresponding to the 1st order static connectivity measures has the lowest value. Since each point in the connectivity pattern is represented by three coordinates, there are exactly three candidates that can be the lowest value coordinate at the first order. On the other hand, the existence of exactly three coordinates for each point in the connectivity pattern is a direct outcome of choosing exactly three electrodes for building the rdFC pattern. This is because three electrodes can combine in only three ways (C(3,2) = 3) with each other when computing all pairwise correlation coefficients among them to obtain the static connectivity measures. Therefore, one can see a direct link between the choice of using three electrodes for pattern generation and the observation of three dominant connectivity patterns. A choice of more than three electrodes will result in more static connectivity measures due to the increase in possible combination pairs. Such a scenario is likely to yield more than three dominant rdFC patterns. However, it is worth noting that such a pattern will have to be appropriately defined because each of its points will have a different number of coordinates owing to the progressively increasing number of connectivity measures for each order.

### Temporal prevalence and evolution of connectivity patterns

To study the temporal prevalence and evolution of rdFC patterns we analyzed long-term EEG records of epilepsy patients in data set 5. Since the EEG recordings often ran into days, we restricted ourselves to just the near set of triplets in this case. We calculated the match percentages of rdFC patterns across time for the EEG records in data set 5. Previously, each epoch was selected from a different subject to calculate the match percentage across subjects within a data set. However, here all the epochs were selected from the same subject to compute the match percentage in a single EEG record across time. The match percentages were above 50% (see Fig. 6a and Supplementary Fig. S2) for most of the triplets and the overall match percentages were above 69% for all the subjects (see Supplementary Table S5) in this data set. The long-term recordings, spanning more than 24 h in all but 5 cases (see Supplementary Table S5), ensured that these patterns were obtained when subjects were in varied states of consciousness. These results suggest that the prevalence of the reference patterns is similar both across time and subjects.

To study the temporal evolution of rdFC patterns we fixed the permutation of the triplets and generated the patterns repeatedly across time in 5-min intervals. In some cases, the match scores with the three references varied substantially across time (Fig. 6b) but for many the patterns consistently matched just one reference pattern throughout the duration of recording (Fig. 6c). The greater prevalence of the latter suggests that for various triplets there is long term stability in their FC as captured by rdFC patterns. However, they were not always stable and in many cases the deviations in the match scores with individual reference patterns coincided with the onset of seizures.

To illustrate this phenomenon clearly, instead of using the three original reference patterns, we obtained two new reference patterns specific to the triplet where such seizure linked transitions in the patterns were occurring. The first reference (preictal) was obtained from the 5-min epoch immediately preceding a seizure, and the second reference (interictal) was obtained from a 5-min epoch 4 hours^34^ prior to the onset of that seizure. The first EEG record of the epilepsy data set (chb_01) had a total of seven seizures and we used the first seizure having a sufficiently long interictal duration (leading seizure) to create the two new references for a triplet displaying the above phenomenon. Taking 5 min of data at a time, we then computed the match scores of this triplet’s chosen permutation with respect to these two references for the entire 40-h recording. We find the match scores with the ictal reference rises above the score with the interictal reference whenever a seizure is about to occur (see Fig. 6d). This suggests that these two connectivity patterns are likely linked with seizure dynamics in this location of the scalp, and characteristically manifest in the EEG during interictal and ictal periods.

## Discussion

Brain network analysis focuses on the topological arrangement of a variable number of nodes, with a fixed notion of edges that connect them. In this study, the emphasis is on multi-order FC of a fixed number of nodes^35^, which reveals a universally recurring correlation structure in human scalp EEG. The correlation structure that is captured by rdFC analysis is expressed through three equivalent connectivity patterns and is characterized by the slopes of the lines in the patterns. The invariance in the slopes ensures that the three correlation coefficients that measure the static functional connectivity of a particular order change in fixed proportions in each successive order. Table 1 and Fig. 4 summarize the prevalence of these characteristic patterns among the 1,070,040 sample patterns obtained from 2356 subjects covering a wide range of EEG variations. Apart from being extensively present within overall data sets, the correlation structure also has a high prevalence within individual EEG records where it is seen at different spatial scales across the scalp.

The recursive nature of rdFC combines multi-scale temporal processing of the underlying neurophysiological data with higher order statistics. One window size worth of data points in a dFC time-series of order *n* requires the use of *n* windows worth of data of the original EEG data. So for a sampling rate of 256 Hz, the first 256 sets (1 window) of connectivity measures in the fifth order time-series are computed using the first 5 × 256 sets (5 windows) of sampled EEG data points. Higher order statistics are inherently computed with the generation of each successive order of dFC due to the recursion of the correlation operation. Use of multi-scale techniques, such as wavelet transforms, is well established in analysis of neurophysiological data^36^. Here the data is examined at a particular scale independently of other scales, whereas rdFC builds a hierarchical graph structure among the various scales as illustrated in Fig. 1c.

Static connectivity measures of nodes obtained via correlation coefficients that are computed with an implicit assumption of stationarity in the activity data are traditionally represented as edges of the outermost triangle in Fig. 1c. This approach can be extended to dFC data of the three nodes in Fig. 1c, which can be thought of as activity data of three pseudo nodes. The static connectivity measures of these pseudo nodes deliver the second order connectivity values that have been shown as the second outermost triangle. More precisely, the first order correlations measure how strongly two nodes are coupled, while second-order correlations measure how closely the dFC of two distinct pairs of nodes are coupled. As shown by the set of nested triangles in Fig. 1c, this recursive coupling can be observed at any order. What our results show is that all these orders of connectivity are found to be interlinked with each other in a manner that is characteristic of scalp EEG.

Though arbitrarily high orders can be computed, we restricted ourselves to the fifth-order. The aim of this study was to ascertain the prevalence of the three reference patterns in larger EEG data sets by pattern matching. It is favorable to have as many orders as possible because it results in a more elaborate connectivity pattern for the purpose of matching. However, we observed two phenomena for larger orders of connectivity due to which the order was restricted to five. These can be seen in Supplementary Fig. S3 where 20 orders have been calculated. The first observation was that connectivity starts with high values near one but as the order increases the values start dropping towards zero, implying low connectivity strength. Secondly, after the fifth order the points follow a repeating spiral pattern tending towards the origin. As a consequence of connectivity values being both small and resulting in a repetitive pattern, any meaningful structure that can be used to compare and distinguish two patterns beyond the fifth order is essentially irrelevant and was, therefore, not considered. In other words, considering more than five orders gives diminishing returns as far as discriminability of the patterns is concerned.

The primary goal of this study was to obtain evidence for the prevalence of a multi-order correlation structure across varied EEG recordings. The variations were sought both in EEG morphologies and underlying neurophysiology, and having subjects of different ages covers both of these aspects well. Electrographic changes in the EEG of neonates occur on a weekly basis^37^ and the interpretation of their EEGs requires such specialized knowledge that neonatal EEG is altogether a separate branch of electroencephalography^38^. The neonate data set stands in contrast with other data sets in this regard and is, therefore, particularly relevant in the exploration of rdFC patterns in a diverse set of EEGs. Like the reference EEG, data set 3 was obtained from healthy adults and the patterns for the near set of triplets were recomputed for it with the sampling rate reduced from 2500 to 250 Hz. Despite sampling rate now matching that of the reference patterns, the match scores were barely affected and the pattern match percentages remained nearly identical across triplets, implying that sampling rates have little bearing on rdFC patterns (see Supplementary Fig. S4).

All the five data sets in this study have a different EEG reference electrode, and sensitivity of connectivity measures on the EEG reference is well known^39,40^. An overall drop of 5% in match percentage was observed when a switch to the average reference electrode was made for the healthy data set (see Supplementary Fig. S4). However, this drop does not necessarily imply that average reference is ill-suited for rdFC analysis since the same is used in data set 4, where it gives results comparable to other data sets. Several neurological conditions are known to have electrographic effects on the EEG^41^ and data set 4 includes many such subjects. This data set is divided into two subsets having normal and abnormal EEG morphologies, respectively. Both subsets consist of EEGs obtained from patients having a wide range of neurological disorders (see Supplementary Table S6). It can be safely assumed that neurophysiologically this data set is far removed from the reference patterns and data set 3, which were both obtained from healthy individuals. The outcome of this study indicates that even in exceedingly disparate data sets the rdFC patterns are present with notable consistency.

Temporal evolution of rdFC patterns in long term EEG recordings of epilepsy patients revealed the presence of the correlation structure even across vigilance states. Importantly, brain states defined by FC have been linked with dynamics around a seizure^42,43^ and we also observed seizure linked changes in connectivity patterns. This can potentially be used in scalp seizure forecasting systems given the practicality of using only three electrodes for processing EEG data for seizure prediction and the robustness of rdFC patterns against artefacts. Using both real and synthetic artefacts, we have earlier shown that robustness to artefacts is an outcome of computing statistical measures over the entirety of EEG epochs that are much longer in duration than typical artefacts^44^. The impact of artefacts of limited duration on static connectivity measures calculated over a 5-min EEG epoch with several thousand samples is substantially mitigated.

To illustrate this for rdFC patterns, we made a copy of the artefact free reference EEG data and introduced two seconds long white Gaussian noise^45^ at every 30-s interval to mimic periodic artefacts. A significant drop in the match score of the pattern obtained from this noise corrupted EEG data only begins to occur as noise power becomes comparable to signal power (see Supplementary Fig. S5). However, the match score settles near 2.2 despite the subsequent increase in noise power because the duration of the artefacts is only a limited portion of the entire 5-min EEG epoch. Though the match score of a sample pattern generated from EEG containing artefacts gets adversely affected, intermittent artefacts that last for a few seconds get “averaged out”. This gives us the robustness needed for making an unbiased selection of epochs across EEG records without worrying about the presence of artefacts.

In this work, functional connectivity is considered in its general form where statistical dependencies are explored in neurophysiological data and not necessarily between brain regions^46^. Consequently, it does not account for volume conduction effects on the correlation structure that are seen on the scalp^47^. It has been suggested the volume conduction effects vary as a function of geodesic distance on the scalp, with electrodes at intermediate distances being the least affected^48^. This was why we employed three sets of triplets with varying mutual separation among the electrodes. Our results show that the characteristic rdFC patterns are robust against change in the distance between the electrodes with consistent match percentages across different triplet sets (see Table 1). However, to develop physiological interpretations based on rdFC patterns requires one to ensure that volume conduction is playing an insignificant role in the analysis, which is not the case in the current study.

A correlation structure that is independent of the mutual separation of the nodes is indicative of spatially scale-invariant FC. Investigations into the functional or structural organization giving rise to such a pervasive multi-order connectivity structure will likely guide future work in this area. Given the widespread presence of the three patterns across neurophysiological, spatial and temporal variations, the correlation structure represented by them can form a criterion for more accurate modeling of electric potentials on the scalp^49^. Apart from providing a method for exploring higher-order individual and group level neuronal dynamics, rdFC captures dynamics of neuronal groups within a single time series that can be used to efficiently study their statistical dependencies. While we calculated higher order functional connectivity of only a triplet of electrodes in this work, inter-triplet functional connectivity can be easily computed to combine an arbitrary number of electrodes and study their higher order dynamics.

## Methods

### Data sets

The details of the datasets used in this study, all of which are publicly available, have been shown in Table 2. One 5-min reference EEG epoch (data set 1) was obtained from the corresponding author. Data set 2 and 3 were acquired from the journal Scientific Data, data set 4 was obtained from Temple University EEG Corpus and data set 5 was taken from the Physionet database. Data set 2 consisted of EEG records of 79 neonates that were acquired from the Helsinki University Hospital^50^. Subjects in data set 3 were healthy adults who were carefully chosen after undergoing an extensive medical and psychological assessment^51^. Data set 4 consists of two groups of subjects who present abnormal and normal EEG morphologies, respectively^52^. Subjects in this data set had prior neurological conditions such as history of seizures, stroke, tumor, trauma, dementia etc., while some were comatose when the EEG was taken (see Supplementary Table S6). Data set 5 consists of long-term EEG records of epilepsy patients who were candidates for surgical intervention^53^. The EEG montage used for one of the patients (chb13) for the majority of the recording period was incompatible for conversion into a montage with common EEG reference for all electrodes, and, therefore, was not considered in our study. In all, EEG records of 2378 unique subjects were considered in this study. In addition to the above, EEG recording of a 25-year old epilepsy patient was used in Fig. 3 to illustrate the methods developed in this work. This and the reference EEG (data set 1) were part of a computational epilepsy biomarker study^44^. The study was approved by the Max Healthcare Ethics Committee of the Super Speciality Max Hospital, Saket and informed consent was obtained from all study participants. All the experiments were performed in accordance with relevant guidelines and regulations.

**Table 2:** Description of data sets used in the study.

Data set	Type	Subjects	Age (median)	Gender (Male/Female)	Reference electrode	Notch frequency (country)	Sampling rate
1	Reference (Healthy)	1	35 years	1/0	Mastoid	50 Hz (India)	250 Hz
2	Neonates	79	36–45 weeks (41 weeks)	42/35	Midline	50 Hz (Finland)	256 Hz
3	Healthy	201	20–35 years (24 years) and 59–77 years (67 years)	128/73	FCz	50 Hz (Germany)	2500 Hz
4a	Abnormal morphology	893	5 days–96 years (55 years)	439/454	Average	60 Hz (USA)	250–512 Hz
4b	Normal morphology	1237	28 days–95 years (44 years)	546/691	Average	60 Hz (USA)	250–512 Hz
5	Long term (Epilepsy)	22	1.5–22 years (10.5 years)	5/16	FT10	60 Hz (USA)	256 Hz

54 subjects are common between data sets 4a and 4b.

### rdFC algorithm and pattern generation

The rdFC algorithm takes multivariate neurophysiological data and computes successive orders of dynamic functional connectivity time-series and static summary connectivity measures using the process illustrated in Fig. 1. To compute rdFC patterns for EEG signals we extract a 5-min long EEG epoch for a triplet of electrodes represented as nodes. For signals sampled at 256 Hz, this translates into a time-series with 5 × 60 × 256 = 76,800 sampled values for each of the three nodes. As the first step, dynamic functional connectivity time-series is generated using the classical sliding window analysis^24^. Here, Pearson correlation coefficients are computed for each window of 1 s duration (256 samples for sampling rate of 256 Hz). Once the computation is made for one window, the window is moved down by just one sample and the correlation coefficients are again computed. Once the window slides through the entire EEG data we obtain the dynamic functional connectivity time-series, which we call the 1st order dFC time-series. On this 1st order dFC time-series, we again perform the entire process of sliding window analysis to obtain the 2nd order dFC connectivity time-series. This process of sliding window analysis is carried recursively four times till we obtain the 4th order dFC time-series.

With five sets of trivariate time-series available, which includes the original EEG data of a triplet of electrodes and four successive dFC time-series, we proceed with the second step. Here the goal is to summarize each trivariate time-series using Pearson correlation coefficients that take into account their entire temporal span. Since there are three variables in each set of time-series, we compute C(3,2) = 3 correlation coefficients for the three possible pairwise combinations of variables in each trivariate time-series. These three correlation coefficients can be regarded as static functional connectivity measures of their respective time-series. The EEG data gives the 1st order static functional connectivity measures. The static connectivity measures computed on the 1st order dynamic functional connectivity time-series give the 2nd order static connectivity measures, and so on until we get the 5th order static connectivity measures from the 4th order dynamic connectivity time-series.

The three measures of static connectivity at each order, lying between − 1 and 1, are represented as three coordinate values of a single point in a 3-dimensional Cartesian coordinate system. Hence, each of the five orders of static functional connectivity can be represented as a point in a three dimensional Cartesian coordinate system, and when connected can be seen as a pattern. Equivalently, each of the three static connectivity measures of each order can be represented by the edges of a triangle. At the first order, the three edges directly connect the three electrodes (nodes) for which the EEG data was processed, and are represented by the outermost triangle in Fig. 1c. The three second order static connectivity measures are represented by the second outermost triangle in Fig. 1c. The other higher order static connectivity measures are represented by the subsequent nested triangles in Fig. 1c.

### Pattern matching algorithm

A pattern matching algorithm was developed to assess the similarity between two arbitrary 3-dimensional 5-point patterns. Upon application of rdFC, each 5-min EEG epoch gives a 5-point pattern representing its five orders of connectivity. The similarity of a given pattern with the reference patterns was measured by comparing their slopes, which ensured the matching was invariant to the size (scale) and location (translation) of the pattern being matched. The vector from the first order point to the second order point was calculated and normalized for the two patterns being compared. Then the dot product of the two vectors was calculated. This was done for all the four vectors in the two rdFC patterns being compared. All the four dot products obtained for each pair of vectors being compared were added, resulting in a total score that can range from − 4 to + 4, with a score of + 4 signifying best match (see pseudocode in Supplementary Fig. S6). Equation (1) is the expression for the match score.

1 $$ \mathop \sum \limits_{i = 1}^{4} \left\langle {a_{i} ,b_{i} } \right\rangle $$

In the above *a*_*i*_ and *b*_*i*_ are the normalized vectors connecting each of the 5 points in the 5-point pattern of the sample pattern being matched and the reference pattern, respectively. They represent the directions of the 4 line segments connecting the 5 points in each pattern. Each pattern being evaluated was matched with all the three reference patterns and the highest score among the three was taken as the match score for the pattern under evaluation.

### Electrode selection and data pre-processing

Although rdFC analysis can be done for any number of nodes, we only performed it using three nodes at a time. The choice of only three electrodes allows us to visualize the outcome of the analysis as a pattern in a 3-dimensional Cartesian coordinate system. Starting with more than a set of three electrodes will generate several summary measures at each order that cannot be easily visualized as points in a higher dimensional space. The choice of three electrodes also keeps the algorithm computationally manageable. For example, starting with 4 electrodes requires computation of more than 1.49 × 10^7^ static connectivity measures due to the combinatorial nature of the algorithm. The total number of correlation pairs generated at each order remains fixed at three if starting with three nodes allowing both the visualization of the resulting patterns and computational tractability.

There are C(16,3) = 560 ways of choosing a triplet of electrodes from the 16 available electrode locations in Supplementary Fig. S1. For computational feasibility three different sets of triplets (near, intermediate, far) were selected with varying levels of mutual separation between the electrode locations (see Supplementary Table S2). The 32 triplets in the near set comprise only of neighboring electrodes while the nodes in the 32 triplets in the intermediate set had a mutual separation of two electrodes. All the nodes in the 10 triplets of the far set were taken from the outermost electrode locations. The triplets were chosen such that the area covered by them on the scalp was maximized.

A raw 5-min EEG epoch was taken and bandpass filtered from 0.5 Hz to 70 Hz to obtain a connectivity pattern. The above frequency range was chosen since it is a global standard^54^ for recording and studying EEG in clinical practice and all the data we have used is from clinical grade EEG machines. Zero-phase filtering was achieved by doing forward and backward filtering for all the data that was processed in this study. The notch filter was placed at 50 Hz for data recorded in India and Europe and at 60 Hz for data recorded in the USA. The choice of window size (1 s) and epoch size (5 min) was in accordance with our previous work^44^ and was maintained across all the calculations performed in this study. The data of the triplet used to build the three reference patterns was obtained from a 5-min epoch that was declared free of artefacts by two trained clinical EEG technicians and two neurologists. Epochs from data sets 2, 3, and 4 were chosen to evaluate connectivity patterns across subjects. We strictly selected the second 5-min EEG epoch from all the records to avoid EEG calibration sequence and eliminate any selection bias. If a flat-line was observed in the data, then the next available epoch was automatically selected. Data set 5 consisted of long-term EEGs of epilepsy patients that were evaluated in their entirety and the durations of each record is presented in Supplementary Table S5. For this data set, the data was transformed to have a common reference electrode for each channel.

### Statistics

A threshold for the match score is needed to determine if an rdFC pattern has a statistically significant match with any of the three reference patterns. The value for this threshold was statistically obtained by randomly generating 100,000 pseudo rdFC patterns. Each correlation coefficient value computed in the rdFC analysis, by definition, lies between − 1 and + 1. An rdFC pattern has three such coefficients computed for each order of correlation, giving a total 15 for all the five orders. A pseudo rdFC pattern was created by generating 15 numbers randomly between − 1 and + 1, with three for each order. All these 100,000 patterns were matched with the three reference patterns using the pattern matching scheme described above and the highest score among the three was selected as the match score. Only five percent of these randomly generated pseudo rdFC patterns had a match score greater than or equal to 2.65. Therefore, for any rdFC pattern to qualify as a match to the three reference patterns, its match score had to be 2.65 or higher.

Two-sided Mann Whitney U test was employed to test the significance of the difference between the two smallest coordinates of the first point in the 5-point connectivity pattern. The two coordinates represent two of the three first order correlation coefficients. The two groups on which the test was performed were the set of patterns that matched exactly one reference (n_1_) and the set of patterns that matched two references (n_2_). The sample populations were drawn from each set of triplet for all the three data sets (data sets 2–4) separately. The choice of using median as the central tendency and Mann–Whitney U test was made due to the presence of substantial outliers. This also obviated the need for applying Fisher transformation on the underlying correlation data since Mann–Whitney U test does not require the sample populations to be Gaussian distributed. Medians and interquartile ranges of all the sample populations whose statistical significance was tested have been reported. Common language effect size, which coincides with the area under the receiver operating characteristic curve, was calculated as U/(n_1_n_2_), where U is the higher Mann Whitney U test statistic.

## Supplementary Information

Supplementary Information.

## Data Availability

All the figures, except Fig. 1, have associated raw data that was drawn from data sets available at: https://zenodo.org/record/2547147#.XcP5G9XhXIU; https://ftp.gwdg.de/pub/misc/MPI-Leipzig_Mind-Brain-Body-LEMON/; https://www.isip.piconepress.com/projects/tuh_eeg/html/downloads.shtml; https://physionet.org/content/chbmit/1.0.0/; https://zenodo.org/record/3547486#.XdPovdXhXIU

## Code availability

The code for generating the 5th order rdFC pattern and the pattern matching algorithm is available at https://github.com/SidPanwar/rdfc.
