# Supplementary material for: Recursive dynamic functional connectivity reveals a characteristic correlation structure in human scalp EEG
Source: Sci Rep. 2021 Feb 2;11:2822. doi: 10.1038/s41598-021-81884-3 (PMC7854737; doi:10.1038/s41598-021-81884-3)
Supplement: Supplementary file 1 — Supplementary Information. [file 41598_2021_81884_MOESM1_ESM.docx]

**Recursive dynamic functional connectivity reveals a characteristic correlation structure in human scalp EEG**

Siddharth Panwar^1*^, Shiv Dutt Joshi^1^, Anubha Gupta^2^, Sandhya Kunnatur^3^ and Puneet Agarwal^4^

^1^ Department of Electrical Engineering, Indian Institute of Technology Delhi, New Delhi 110016, India.
^2^ Department of Electronics and Communication Engineering, Indraprastha Institute of Information Technology, New Delhi 110020, India
^3^ Arghya Bioinformatics, New Delhi, India ^4^ Max Super Speciality Hospital, Saket, New Delhi 110017, India
^*^[siddharthpanwar@alumni.stanford.edu](mailto:siddharthpanwar@alumni.stanford.edu)


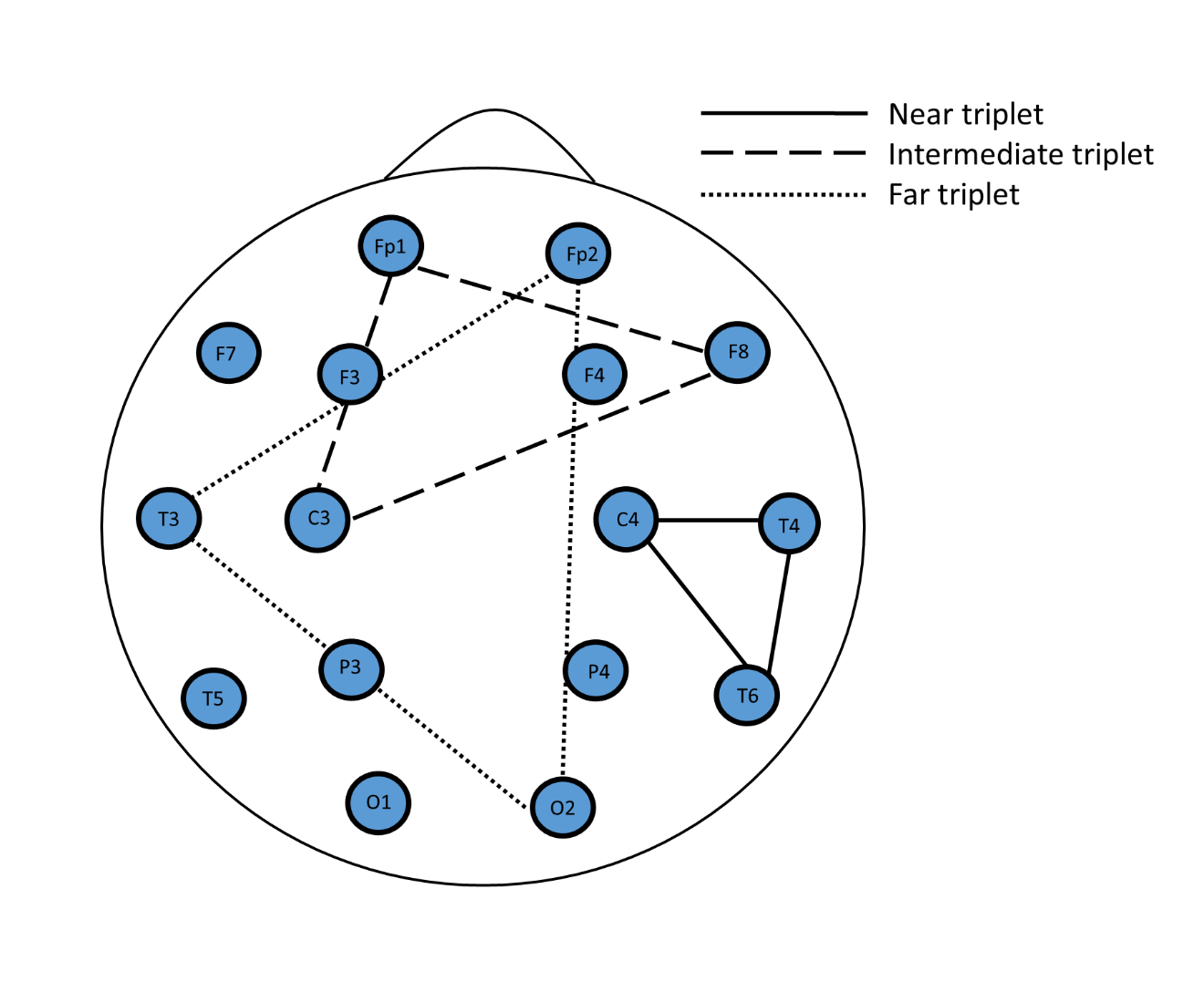


Supplementary Fig. S1 Electrode locations on the scalp used to select the three sets of triplets, with an example from each set, namely, near (solid line), intermediate (dashed line) and far (dotted line) shown.


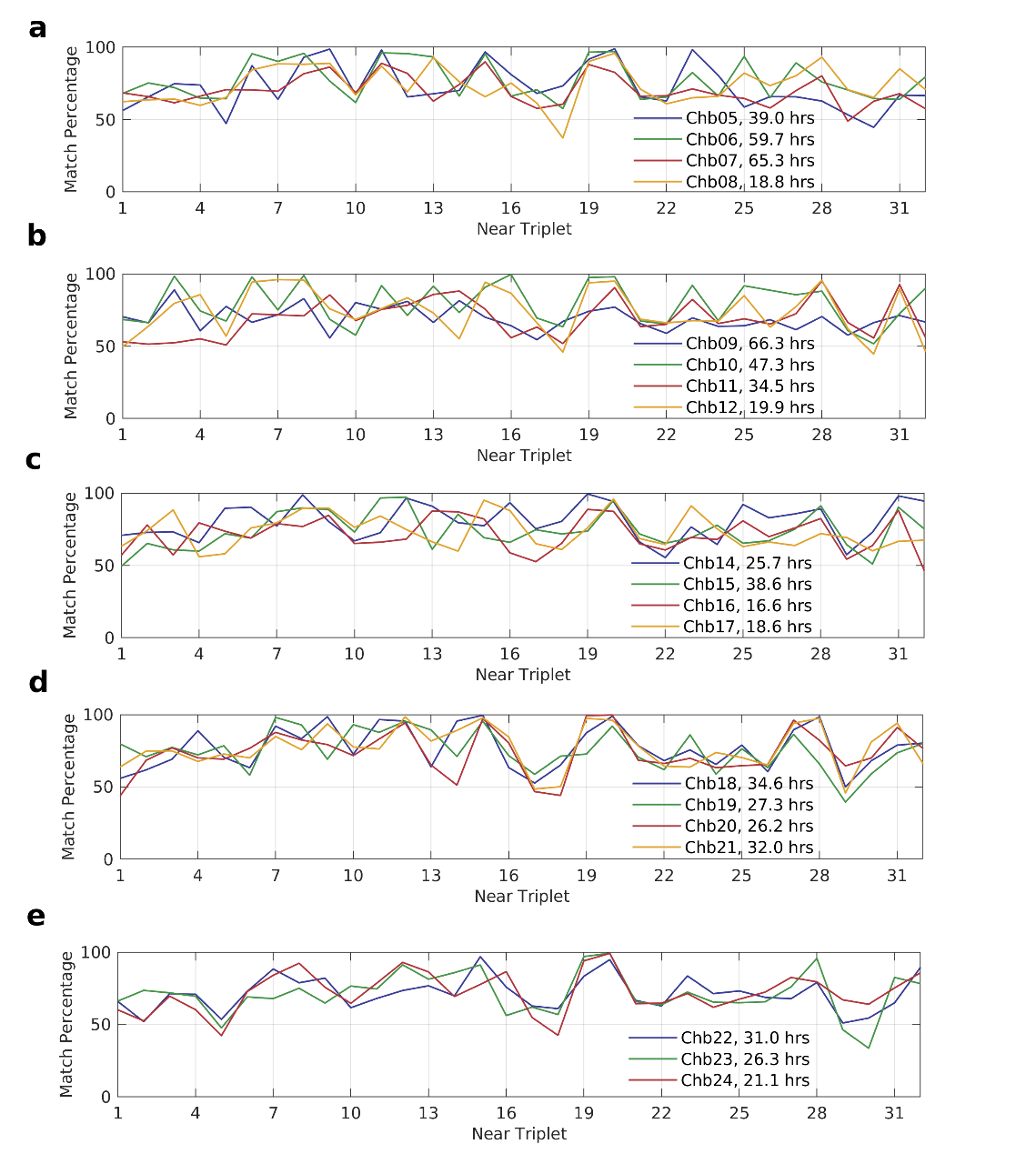


Supplementary Fig. S2 Match percentage of 19 EEG records from Chb05 to Chb24 (except Chb13) in data set 5 across all six permutations of each triplet.


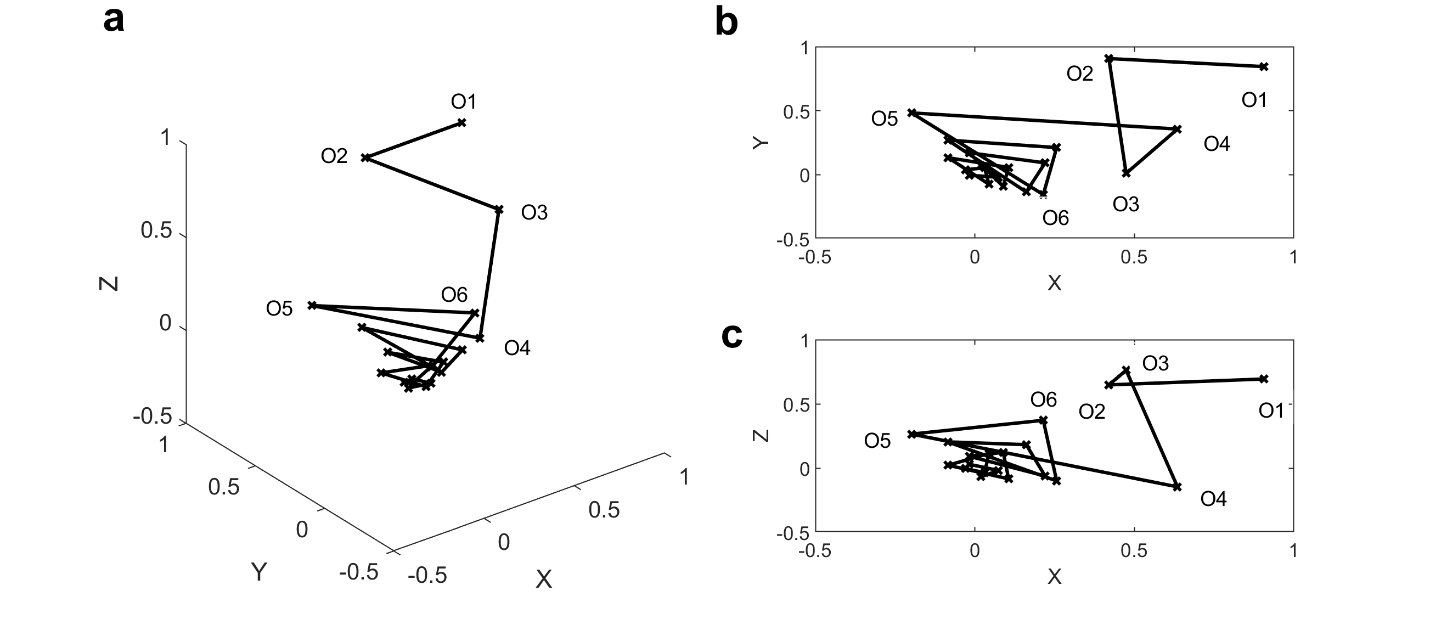


Supplementary Fig. S3 Recursive dynamic connectivity pattern computed over 20 orders of correlation coefficients. **a** Reference 1 with the projection of the pattern shown in **b** X-Y and **c** X-Z planes.


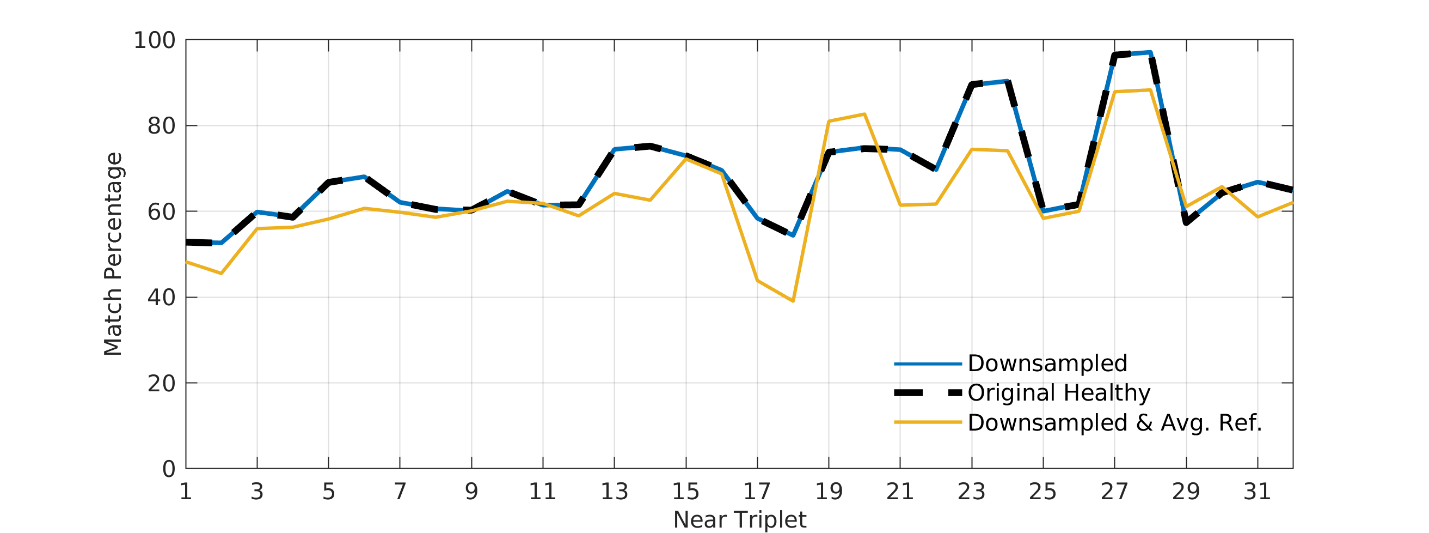


Supplementary Fig. S4 Match percentage of healthy subjects when EEG signals are downsampled from the original 2500 Hz to 250 Hz and then also with EEG reference changed from FCz in the original data set to average reference.


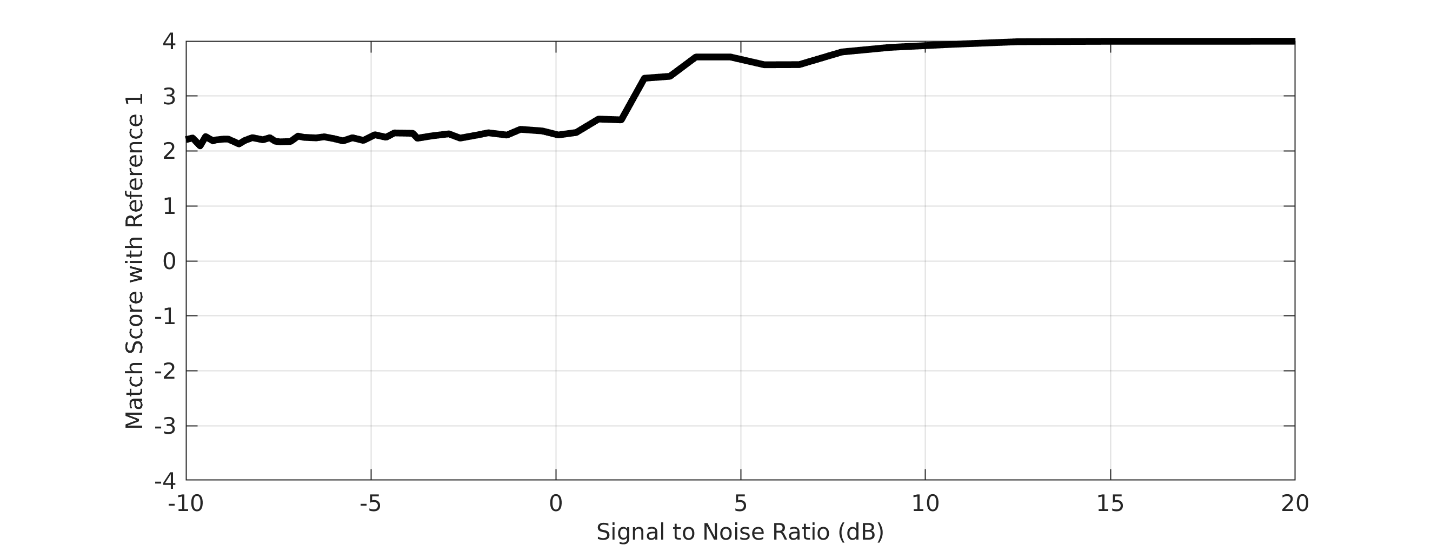


Supplementary Fig. S5. Impact of synthetic artifacts on match score. A copy of the original artifact free EEG reference data (data set 1) was made and synthetic artifacts, modeled as white Gaussian noise, were added independently on all three electrode signals of the triplet. Two seconds of noise was added at every 30 second interval, totaling 20 seconds for the entire 5-minute EEG epoch. The permutation of the reference data triplet used to evaluate the impact of the added noise was the one that gives reference 1. Noise power was gradually increased to reduce the signal to noise ratio in the copy of the reference data and corresponding match scores with the original reference were computed.


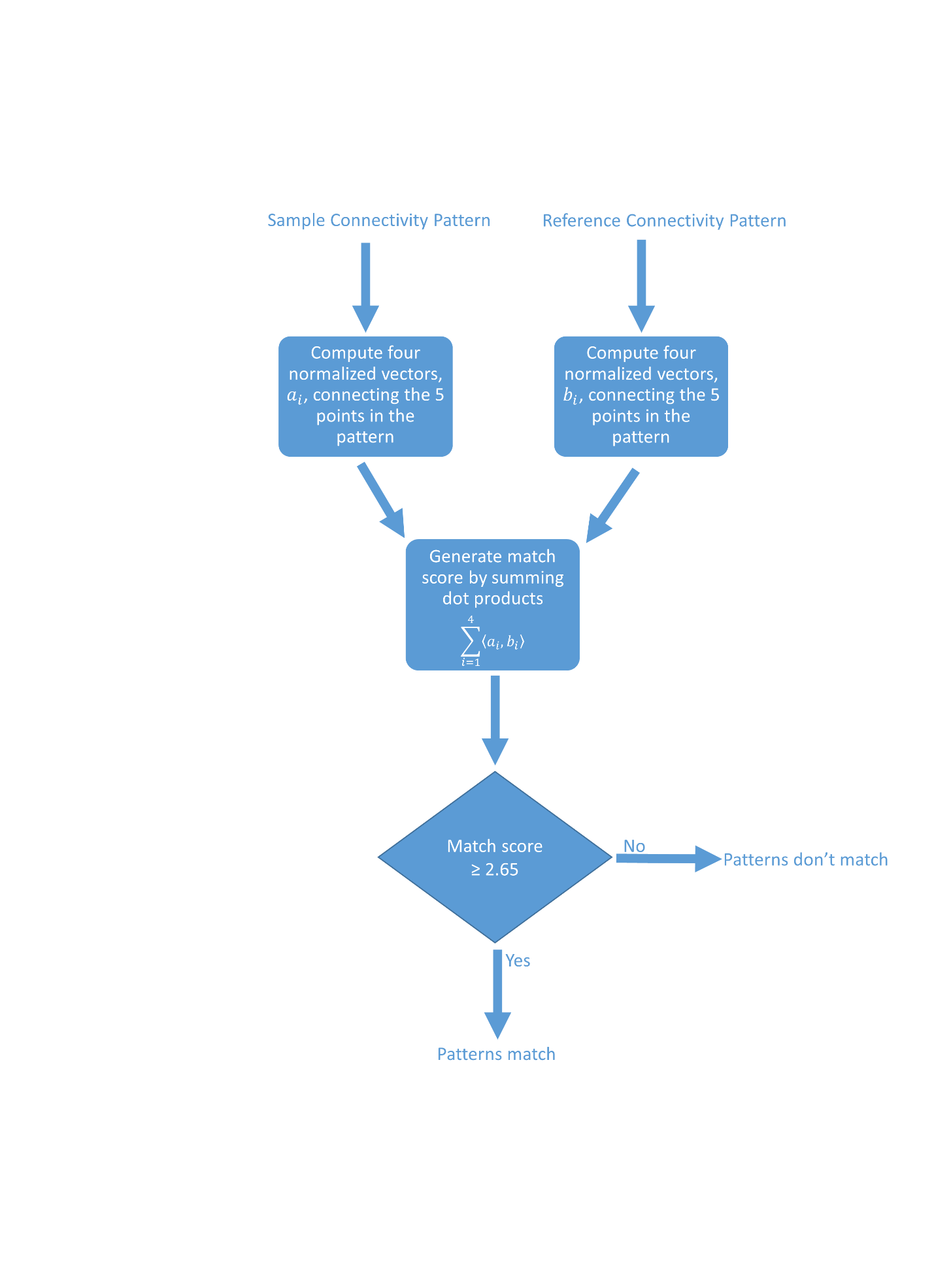


Supplementary Fig. S6. Pseudocode for the pattern matching algorithm.

Supplementary Table S1. Change in the sequence of coordinates of each point in the 5-point connectivity pattern for different permutations of a given triplet

| Permutation | Coordinate | Order 1 | Order 2 | Order 3 | Order 4 | Order 5 |
| --- | --- | --- | --- | --- | --- | --- |
| (C3,T3,T5) | $x$ | x_1_ | x_2_ | x_3_ | x_4_ | x_5_ |
|  | $y$ | y_1_ | y_2_ | y_3_ | y_4_ | y_5_ |
|  | $z$ | z_1_ | z_2_ | z_3_ | z_4_ | z_5_ |
| (C3,T5,T3) | $x$ | z_1_ | y_2_ | x_3_ | z_4_ | y_5_ |
|  | $y$ | y_1_ | x_2_ | z_3_ | y_4_ | x_5_ |
|  | $z$ | x_1_ | z_2_ | y_3_ | x_4_ | z_5_ |
| (T3,C3,T5) | $x$ | x_1_ | z_2_ | y_3_ | x_4_ | z_5_ |
|  | $y$ | z_1_ | y_2_ | x_3_ | z_4_ | y_5_ |
|  | $z$ | y_1_ | x_2_ | z_3_ | y_4_ | x_5_ |
| (T3,T5,C3) | $x$ | y_1_ | y_2_ | y_3_ | y_4_ | y_5_ |
|  | $y$ | z_1_ | z_2_ | z_3_ | z_4_ | z_5_ |
|  | $z$ | x_1_ | x_2_ | x_3_ | x_4_ | x_5_ |
| (T5,C3,T3) | $x$ | z_1_ | z_2_ | z_3_ | z_4_ | z_5_ |
|  | $y$ | x_1_ | x_2_ | x_3_ | x_4_ | x_5_ |
|  | $z$ | y_1_ | y_2_ | y_3_ | y_4_ | y_5_ |
| (T5,T3,C3) | $x$ | y_1_ | x_2_ | z_3_ | y_4_ | x_5_ |
|  | $y$ | x_1_ | z_2_ | y_3_ | x_4_ | z_5_ |
|  | $z$ | z_1_ | y_2_ | x_3_ | z_4_ | y_5_ |

The triplet (C3,T3,T5) has been used merely as an example to illustrate the six permutations and the above applies equally to all triplets. The coordinates representing each point of the 5-point connectivity pattern for the first permutation are (x_n_, y_n_, z_n_), where n denotes the order. The coordinates for the 5-point connectivity patterns of the remaining 5 permutations are a rearrangement of the sequence of the coordinates of the first permutation.

Supplementary Table S2. The three sets of electrode triplets grouped in accordance with the mutual separation of electrodes

| Triple | Near | Intermediate | Far |
| --- | --- | --- | --- |
| 1 | Fp1 F7 F3 | Fp1 T3 C4 | T3 Fp2 T6 |
| 2 | Fp2 F8 F4 | Fp2 T4 C3 | T4 Fp1 T5 |
| 3 | Fp1 F3 F4 | Fp1 C3 T4 | T3 O2 F8 |
| 4 | Fp2 F4 F3 | Fp2 C4 T3 | T4 O1 F7 |
| 5 | F7 T3 C3 | F7 T5 P4 | T3 F8 T6 |
| 6 | F8 T4 C4 | F8 T6 P3 | T4 F7 T5 |
| 7 | F7 F3 C3 | F7 F4 P3 | Fp1 T5 T6 |
| 8 | F8 F4 C4 | F8 F3 P4 | Fp2 T5 T6 |
| 9 | F3 F7 T3 | F3 T5 P4 | O1 F7 F8 |
| 10 | F4 F8 T4 | F4 T6 P3 | O2 F7 F8 |
| 11 | F3 C3 T3 | F3 P3 F8 | - |
| 12 | F4 C4 T4 | F4 P4 F7 | - |
| 13 | F3 C3 C4 | T3 Fp2 P4 | - |
| 14 | F4 C4 C3 | T4 Fp1 P3 | - |
| 15 | F4 F3 C3 | T3 F4 O2 | - |
| 16 | F3 F4 C4 | T4 F3 O1 | - |
| 17 | C3 T3 T5 | T5 F7 F4 | - |
| 18 | C4 T4 T6 | T6 F8 F3 | - |
| 19 | C3 P3 T5 | T5 Fp1 P4 | - |
| 20 | C4 P4 T6 | T6 Fp2 P3 | - |
| 21 | T3 C3 P3 | O1 T3 C4 | - |
| 22 | T4 C4 P4 | O2 T4 C3 | - |
| 23 | T3 T5 P3 | O1 C3 T4 | - |
| 24 | T4 T6 P4 | O2 C4 T3 | - |
| 25 | C4 C3 P3 | C3 Fp2 O2 | - |
| 26 | C3 C4 P4 | C4 Fp1 O1 | - |
| 27 | C3 P3 P4 | C3 F8 T6 | - |
| 28 | C4 P4 P3 | C4 F7 T5 | - |
| 29 | O1 T5 P3 | P3 F3 T6 | - |
| 30 | O2 T6 P4 | P4 F4 T5 | - |
| 31 | O1 P3 P4 | P3 F3 T6 | - |
| 32 | O2 P4 P3 | P4 F7 F8 | - |

Supplementary Table S3. Statistical significance test for showing that the difference between the two lowest coordinate values of the first order point of the 5-point connectivity patterns matching one reference pattern (n_1_) is greater than that of those matching two reference patterns (n_2_).

| Data set | | Near | Intermediate | Far |
| --- | --- | --- | --- | --- |
| Neonates | Mann-Whitney U | 2162694 | 1850200 | 206450 |
|  | Effect size | 0.73 | 0.71 | 0.65 |
|  | p-value | 2.36 × 10^-108^ | 7.72 × 10^-77^ | 4.30 × 10^-15^ |
|  | Sample size (n_1_,n_2_) | (9714, 828) | (8901, 713) | (2375, 249) |
| Healthy | Mann-Whitney U | 10267536 | 11776353 | 3468645 |
|  | Effect size | 0.79 | 0.76 | 0.62 |
|  | p-value | 0 | 0 | 4.30 × 10^-46^ |
|  | Sample size (n_1_,n_2_) | (24179, 2058) | (22241, 2187) | (5900, 1541) |
| Abnormal | Mann-Whitney U | 196115420 | 131091422 | 24047450 |
|  | Effect size | 0.78 | 0.79 | 0.72 |
|  | p-value | 0 | 0 | 0 |
|  | Sample size (n_1_,n_2_) | (109733, 8264) | (112823, 5667) | (31678, 2715) |
| Normal | Mann-Whitney U | 342372507 | 243825079 | 50386479 |
|  | Effect size | 0.78 | 0.78 | 0.70 |
|  | p-value | 0 | 0 | 0 |
|  | Sample size (n_1_,n_2_) | (150278, 41552) | (153166, 7237) | (41552, 4018) |

Supplementary Table S4. Difference between the two lowest coordinates (correlation coefficients) of the first order point of the 5-point pattern of matching patterns

| Dataset | Median difference [interquartile range] for patterns matching one reference (number of samples, n_1_) | | | Median difference [interquartile range] for patterns matching two references (number of samples, n_2_) | | |
| --- | --- | --- | --- | --- | --- | --- |
|  | Near | Intermediate | Far | Near | Intermediate | Far |
| Neonates | 0.09 [0.04-0.18]  (9714) | 0.10 [0.04-0.18]  (8901) | 0.07 [0.03-0.13]  (2375) | 0.04 [0.01-0.07]  (828) | 0.04 [0.02-0.08]  (713) | 0.04 [0.02-0.07]  (249) |
| Healthy | 0.14 [0.07-0.24]  (24179) | 0.16[0.08-0.26]  (22241) | 0.07 [0.03-0.13]  (5900) | 0.04 [0.02-0.08]  (2058) | 0.06 [0.03-0.11]  (2187) | 0.04 [0.02-0.08]  (1541) |
| Abnormal | 0.11 [0.05 - 0.21]  (109733) | 0.17 [0.08-0.29]  (112823) | 0.11 [0.5-0.19]  (31678) | 0.03 [0.01 - 0.06]  (8264) | 0.05 [0.02-0.1]  (5667) | 0.05 [0.02-0.09]  (2715) |
| Normal | 0.11 [0.05 - 0.21]  (150278) | 0.16 [0.08-0.26]  (153166) | 0.09 [0.04-0.16]  (41552) | 0.03 [0.01 - 0.06]  (41552) | 0.05 [0.02-0.09]  (7237) | 0.04 [0.02-0.08]  (4018) |

Supplementary Table S5. Overall match percentages of all six permutations of the near set of triplets for records in data set 5.

| Patient ID | Overall percentage match (%) | Duration (Hours) |
| --- | --- | --- |
| Chb01 | 75.1 | 38.6 |
| Chb02 | 74.7 | 35.2 |
| Chb03 | 72.4 | 37.1 |
| Chb04 | 71.2 | 141.4 |
| Chb05 | 72.8 | 39.0 |
| Chb06 | 77.5 | 59.7 |
| Chb07 | 70.1 | 65.3 |
| Chb08 | 73.9 | 18.8 |
| Chb09 | 69.2 | 66.3 |
| Chb10 | 79.4 | 47.3 |
| Chb11 | 69.3 | 34.5 |
| Chb12 | 74.0 | 19.9 |
| Chb14 | 80.6 | 25.7 |
| Chb15 | 74.0 | 38.6 |
| Chb16 | 71.5 | 16.6 |
| Chb17 | 73.3 | 18.6 |
| Chb18 | 77.1 | 34.6 |
| Chb19 | 75.5 | 27.3 |
| Chb20 | 74.0 | 26.2 |
| Chb21 | 77.2 | 32.0 |
| Chb22 | 71.7 | 31.0 |
| Chb23 | 71.5 | 26.3 |
| Chb24 | 72.3 | 21.1 |

Supplementary Table S6. Subjects with a prior diagnosis of a neurological condition in data set 4

| Neurological disorder | Abnormal  Morphology | Normal  Morphology |
| --- | --- | --- |
| History of seizures | 493 | 648 |
| Trauma | 51 | 70 |
| Stroke | 195 | 87 |
| Migraine | 5 | 69 |
| Tumor | 34 | 15 |
| Hydrocephalus | 7 | 3 |
| Cerebral Palsy | 6 | 9 |
| Dementia | 35 | 11 |
| Multiple Sclerosis | 7 | 4 |
| Parkinson’s disease | 1 | 5 |
| Coma | 30 | 0 |
